# IMU-based classification of resistive exercises for real-time training monitoring on board the international space station with potential telemedicine spin-off

**DOI:** 10.1371/journal.pone.0289777

**Published:** 2023-08-10

**Authors:** Martina Ravizza, Laura Giani, Francesco Jamal Sheiban, Alessandra Pedrocchi, John DeWitt, Giancarlo Ferrigno

**Affiliations:** 1 NearLab, Department of Electronics, Information and Bioengineering, Politecnico di Milano, Milano, Italy; 2 Independent Researcher, Italy; Polytechnic University of Marche: Universita Politecnica delle Marche, ITALY

## Abstract

The microgravity exposure that astronauts undergo during space missions lasting up to 6 months induces biochemical and physiological changes potentially impacting on their health. As a countermeasure, astronauts perform an in-flight training program consisting in different resistive exercises. To train optimally and safely, astronauts need guidance by on-ground specialists via a real-time audio/video system that, however, is subject to a communication delay that increases in proportion to the distance between sender and receiver. The aim of this work was to develop and validate a wearable IMU-based biofeedback system to monitor astronauts in-flight training displaying real-time feedback on exercises execution. Such a system has potential spin-offs also on personalized home/remote training for fitness and rehabilitation. 29 subjects were recruited according to their physical shape and performance criteria to collect kinematics data under ethical committee approval. Tests were conducted to (i) compare the signals acquired with our system to those obtained with the current state-of-the-art inertial sensors and (ii) to assess the exercises classification performance. The magnitude square coherence between the signals collected with the two different systems shows good agreement between the data. Multiple classification algorithms were tested and the best accuracy was obtained using a Multi-Layer Perceptron (MLP). MLP was also able to identify mixed errors during the exercise execution, a scenario that is quite common during training. The resulting system represents a novel low-cost training monitor tool that has space application, but also potential use on Earth for individuals working-out at home or remotely thanks to its ease of use and portability.

## Introduction

Prolonged exposure to microgravity during long term spaceflights has been addressed as one of the main stress factors for the astronauts, as it is responsible for several physiological alterations affecting mostly the cardio-vascular and musculoskeletal systems, with consequences getting worse as the time of the mission increases. Over the last decades of space explorations, Long Duration Mission (LDM) allowed to collect data indicating a loss of bone mineral density of 5%, a reduction of muscle mass up to 35–40% and cardiovascular functions alteration [1]. To reduce these health problems, several countermeasure training programs have been implemented. The protocol considered in this paper is based on resistive exercises performed with the Advance Resistive Exercises Device (ARED) currently used on the International Space Station (ISS), which allows astronauts to perform typical gym exercises. Currently, ISS crew-members receive feedback from on ground specialists by using a real-time audio/video system to ensure optimal and safe performance [2]. However, as the distance from the Earth to the space vehicle increases, communication delays increase and loss of communication can occur. Consequently, training monitoring during future planned LDMs to the Moon and Mars could be problematic. It has been assessed that non-optimal exercise performance, especially by using high loads, may reduce training efficacy and can involve risk of injuries [3–5]. To overcome reduced opportunity for human coaching, the introduction of motion tracking technologies could be useful.

Several studies in literature used motion capture systems to analyze motion during training exercises [6–9] and movement in microgravity conditions [10–14]. These solutions are not suitable for real-time monitoring on the ISS since they require bulky technology and complex system setup and operation. Conversely, Inertial Measurement Units (IMUs) are small and inexpensive devices that can be used to quantify human motion. IMUs have indeed been used for daily human activities recognition [15–17] as well as gait analysis [18], elderlies fall detection [19], medical monitoring [20] and stereotypical motor movements recognition in autism spectrum disorder [21].

Data from IMUs can also be used to classify typical gym exercises with supervised learning methods [22, 23]. Lee *et al*. [24] compared conventional machine learning (CML) and deep learning (DL) algorithms for detecting five induced deviations of squat by using five IMUs placed on the body. They obtained accuracies equal to 75.4% for CML and 91.7% for DL. Similarly, O’Reilly *et al*. [25] classified five induced deviations of deadlift performance starting from data collected by five IMUs with accuracies of 60% and 81%, respectively. Other studies used data collected from inertial sensors to classify various types of exercises performed in the same experimental session. De Villa *et al*. [26] used four IMUs to classify a set of seven exercises of upper and lower limbs frequently proposed in physical therapy routines (including squats, hip abduction, knee flex-extension, gait, elbow flex-extension, extension of arms overhead and squeezing), obtaining sensitivity and specificity values over 99% in the detection of wrongly performed motions using a Support Vector Machine (SVM) with a polynomial kernel. Depari *et al*. [27] collected data from a single wrist-worn wearable IMU of fourteen participants and classified a subset of the same exercises with Linear Discriminant Analysis (LDA), obtaining average exercise detection accuracy above 93% and errors in exercise repetitions counting less than 6%. Finally, Preatoni *et al*. [28] monitored the execution of four type of functional workout exercises (Clean and Jerk, Burpee, American Swing, Box Jump) using five IMUs located on the upper and lower limb, and on the trunk of fourteen participants using both k-Nearest Neighbours (kNN) algorithms with different types of metrics and SVM with several type of kernel functions. The authors carried out a sensitivity analysis testing the performance of the two classifiers with different parameters combinations, and selecting data from different subset of the five IMUs available, showing a range of accuracy from 82.5% to 97.8%.

However, while these works represent efficient on-Earth training monitoring approaches, they present three additional issues for space applications. The first is that acceleration data includes gravity component, not consistent with a microgravity scenario. The second is that the magnetometer signals (needed to perform the classification) are much different on the ISS since its magnetic field quickly changes in time. Finally, exercises were performed without any additional external load, which may influence movement kinematics.

The aim of this work was to design a wearable IMU system and to develop and validate an algorithm for classifying resistance training. Key aspects were: (a) suitability of use in microgravity; (b) portability, wearability and easiness to use; (c) capability to provide real-time instructions and corrective feedback also personalized to the single individual.

This tool could also have a potential extended application for individual training or rehabilitation at home, as our system may help to perform home workout safely and without local human supervision.

## Materials and methods

### Measuring system

**Fig 1:**
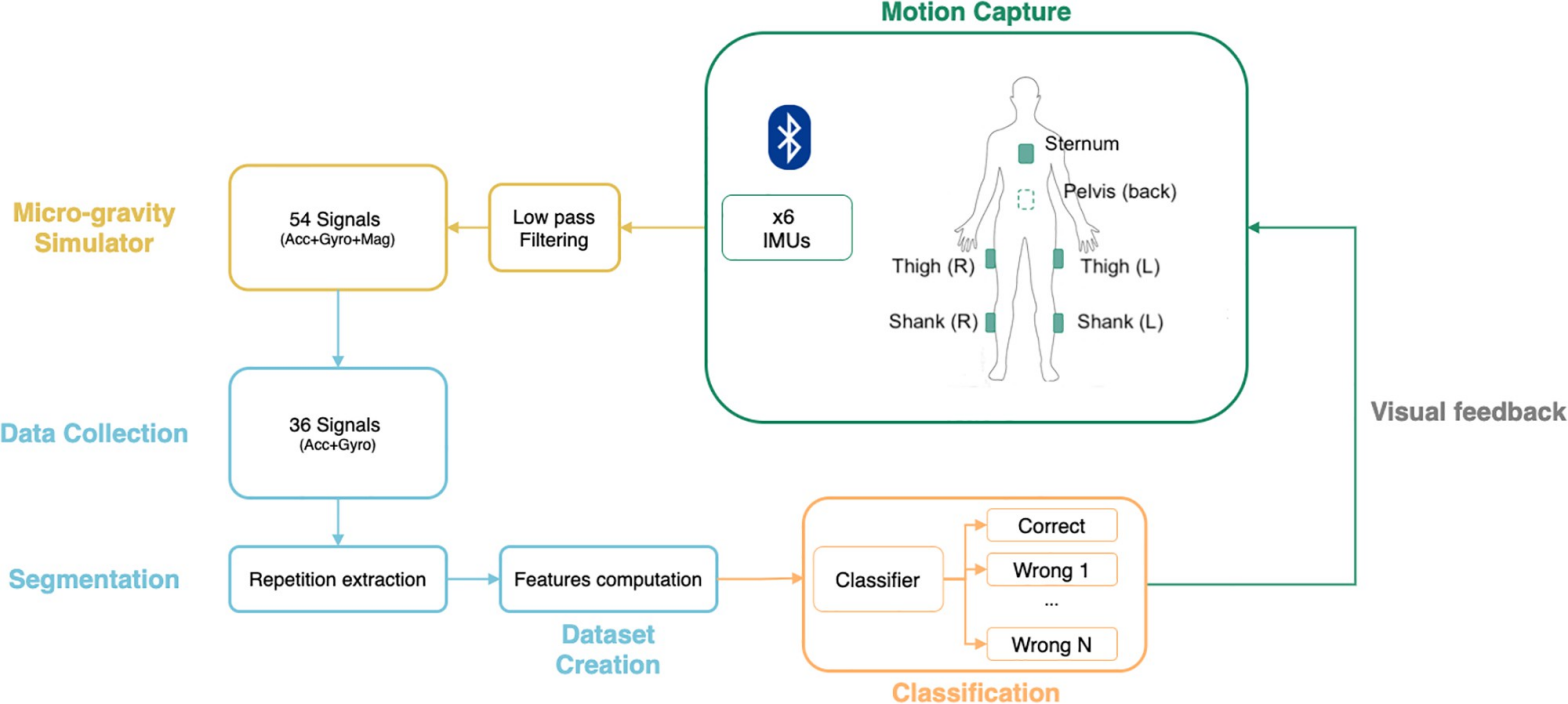
Real-time biofeedback system diagram. Schematic representing the real-time biofeedback system: the gravitational component of the filtered signals collected from the six IMUs placed on the subject’s body is removed using the magnetometers’ data. Then, the resulting accelerometers and gyroscopes signals are segmented and organised in a dataset by extracting the most informative set of features (such as mean, standard deviation, mean frequency, temporal entropy, etc.) for each x-y-z temporal serie of each IMU. The dataset is used to train a ML classifier to monitor the execution of different resistive exercises and give a real-time visual feedback to the subject undergoing training through a GUI.

The real-time biofeedback system (Fig 1) developed was composed by six IMUs, specially designed for this work and named SpaceSens (see hardware section below), which collect data synchronously and send them via Bluetooth to a processing device. In the latter (see Signal Processing section below), the signals are processed in order to simulate data acquired in microgravity, to extract a single gesture repetition and to perform error classification. A simple Graphical User Interface (GUI) allows users to easily start the tool and delivers visual biofeedback during training.

#### Hadware

The SpaceSens IMUs include a magnetic-inertial sensor, a microcontroller, a communication system and an integrated power supply. Each component is electrically connected through a Printed Circuit Board (PCB) and embedded in a 3D printed case. The SparkFun MPU-9250 IMU Breakout was chosen as inertial sensor. It is made up of 3-axis accelerometer, 3-axis gyroscope and 3-axis magnetometer. Arduino Pro Mini 328 microcontroller has been used to communicate with a Personal Computer (PC) through a Low Energy Bluetooth HC-05 module, ensuring low power consumption and small size requirements (13x26.9x2.2 mm). Power supply is provided by a rechargeable Li-Po battery with a capacity of 750 mAh that ensures a working time of 18 hours. The final device implemented measures 61x61x33.5 mm, weighs 80g and guarantees an output rate of 100 Hz.

#### Signal processing

**Fig 2:**
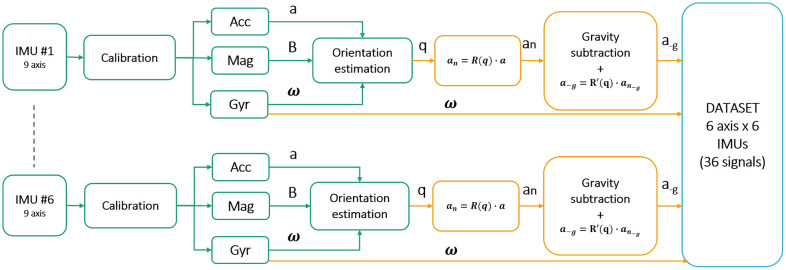
Signal processing workflow. Schematic describing the method used to obtain the final dataset composed by 36 signals, including accelerations cleaned from gravity component and angular velocities of the six IMUs.

The nine signals of each IMU were initial filtered with a Low Pass filter with a cut-off frequency of 20 Hz to remove high-frequency noise. Microgravity conditions were simulated by estimating IMU orientations through the Mahony complementary filter [29] to project acceleration data on the Earth RF and subtract the gravitational component, and expressing the resulting quantities back to the sensor RF. The processed dataset was composed of six signals (Fig 2) and used as input to the classification algorithm.

#### Graphical user interface

The GUI was created with the Python Tkinter toolkit, and included all the elements to connect the PC to the IMUs and to visualise and store the data recorded. Moreover, it permitted to start the monitoring and/or the data collection.

### Experimental protocol

#### Participants

The Politecnico di Milano Ethics Committee approved this study (approval n. 34/2020) and a written informed consent was signed by participants before the experiments. Data collection was performed during two separate sessions by recruiting a total of 29 subjects with the following inclusion criteria: healthy, trained, with previous experience of weightlifting, with no musculoskeletal injuries and aware of their strength capacity, computed with the ‘one repetition maximum’ test.

#### IMUs placement

**Fig 3:**
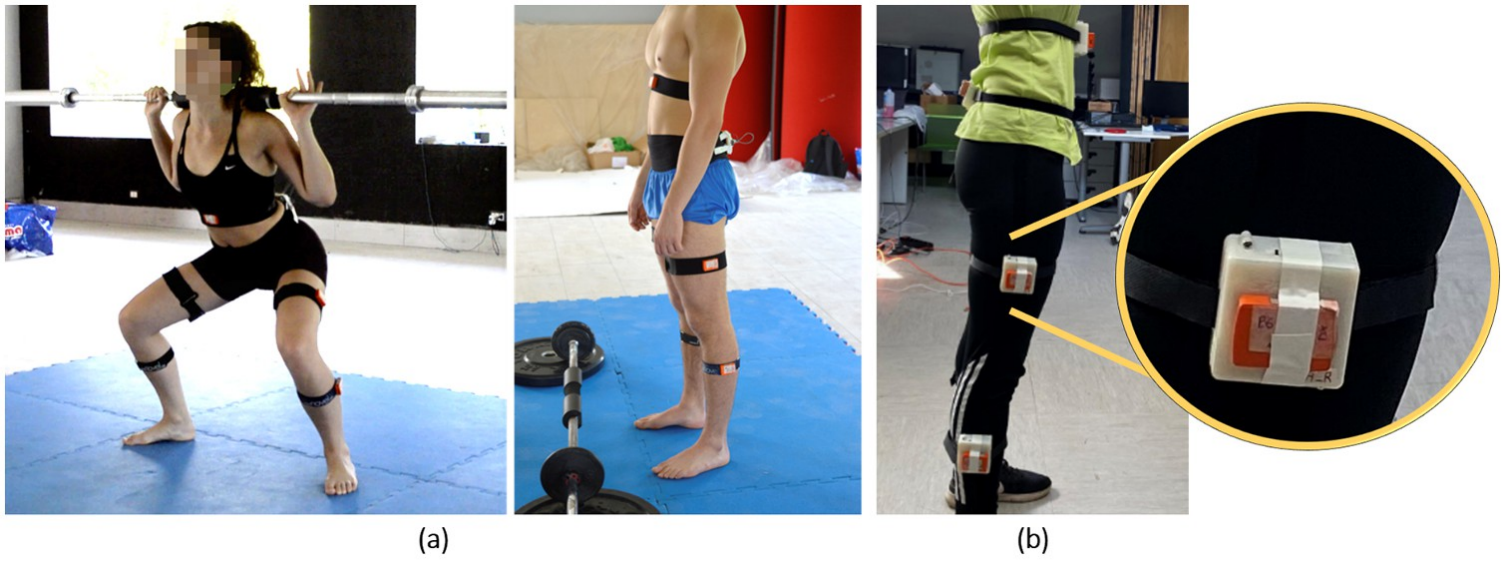
IMUs placement during the experiments. (a) First session of data collection with Xsens IMUs; (b) second session of acquisition with the two sensors superimposed.

During the first data collection session, 17 subjects (9 Males, 26.89 ± 5.73 years old, 64.22 ± 7.14 kg, 173,44 ± 4.25 cm; 8 Females, 25.38 ± 3.77 years old, 56 ± 6.31 kg, 163.14 ± 6.52 cm height) wore five MTw Awinda IMUs (Xsens Technologies, Enschede, Netherlands) fixed with elastic bands to each shank (medial point between lateral malleolus and lateral femoral condyle), each thigh (medial point between lateral femoral condyle and greater trochanter), over the sacrum (centrally between the two posterior superior iliac spines) and over the sternum (Fig 3a). External load was applied using an Olympic barbell and weights. The second session, involving the remaining 12 subjects (8 Males, 25.5 ± 0.7 years old, 71.5 ± 16.26 kg, 179,5 ± 6.36 cm; 4 Females, 26 ± 1.4 years old, 62 ± 11.31 kg, 167 ± 4.24 cm height), aimed to test the real-time classification. Additionally, 4 of those 12 subjects, wore SpaceSens superimposed to Xsens IMUs, on the same body points of the first experiment (Fig 3b), in order to validate SpaceSens by comparing signals. Each couple of sensors (SpaceSens and Xsens) were fixed by aligning axis and by using adhesive tape.

Note that, although the subjects performed the exercises using a freely-moving barbell (as opposed to the ARED device that mechanically constrain the jittering movement of the weight during squats and deadlift), our previous work [6] compared kinematics data collected from capturing the body motion of subjects preforming exercises using the on-ground ARED mockup at Johnson Space Center (JSC) in Houston, Texas (US) NASA and using a barbell, and confirmed via a Wlicoxon-Mann-Whitney test (*p* < 0.05) the signals correlation and the equivalence of joint angles in the sagittal plane.

#### Exercises

After a self administered warm-up of about 10–15 minutes, each participant was instructed to complete 5 to 20 repetitions of the parallel squat exercise. The squats were performed with different techniques in six separated trials. In particular, the first trial required a proper execution of the exercise [5] and the other five trials included selected mistakes that were defined as the most common and dangerous, in accordance with the NASA Astronaut Strength, Conditioning and Rehabilitation (ASCR) specialists at JSC. Classes of incorrectness were: rounded back (RB), knees overcoming toes (KOT), valgus knees (VK), raised heels (RH) and shallow squat (SH).

### Classification

#### Pre-processing and segmentation

A low pass 6th order Butterworth filter (2 Hz cut-off frequency) was applied to the acceleration data with the gravitational vector removed and to angular velocities obtained from the IMUs. Then, a peak and valley detection algorithm was applied to automatically extract each repetition, by identifying the starting points and the ending points as the zero-crossings before each peak and after each valley, respectively. For each of the six sensors, two dataset tables were created, one containing x, y and z 0g accelerations entries and another including the corresponding components of angular velocity.

#### Feature extraction and dataset creation

Features in time and frequency domains were computed from the segmented signals of each sensor. The 2250 features, 375 per sensor, were: mean, standard deviation (SD), median absolute deviation (MAD), maximum, minimum, signal magnitude area (SMA), energy, interquartile range (IQR), entropy in both domains; autoregressive model coefficients and correlation coefficient for normal and jerk signals in time domain; index of first occurrence of maximum (maxInds), mean frequency, skewness and kurtosis for frequency signals. Feature scaling was then performed by using a Robust Scaler [30] and the dataset was reduced to increase efficiency of the classifier by applying the Recursive Feature Elimination (RFE) method [31]. The ideal number was chosen by applying RFE multiple times and considering the one that permitted to reach the best results of the classification. The resulting dataset was then used to train and test the classifiers, considering 70% and 30% of all the values respectively.

#### Classification algorithms

Five supervised machine learning methods were compared: Decision Trees, Random Forest, K-Nearest Neighbour (KNN), Support Vector Machine (SVM) and Multi-Layer Perceptron (MLP). Each algorithm was trained and tested with the same subset of data and their performances were evaluated by a stratified 10-fold Cross-Validation. Accuracy (see Eq 1 below, where TP indicates True Positive, TN True Negative, FP False Positives, FN False Negatives, and k is the summation index over the classes) was used to evaluate and compare the performances of the models as it reflects the portion of correct classified observations.

#### Real-time system testing

After evaluating the classifiers’ performance, we conducted a follow-up session (namely, the second session reported in the experimental protocol description above) in which the best classifier was tested with real-time data collected from the participants and transmitted via Bluetooth to the software connected the sensors. The classification result was displayed on screen via the GUI for immediate feedback, and the provided outcomes were also stored to compute the classifier’s performance (see Table 3 in Results).

### Statistical analysis

#### Sensor validation

To validate SpaceSens performance, we compared it with the state-of-the-art inertial sensors Xsens MTw Awinda. We compared signals of acceleration, angular velocity and acceleration cleaned of gravity by a pre-processing step followed by the computation of statistical values in time and frequency domains. Signals were first filtered with a 6th order low-pass Butterworth filter at 2Hz to exclude high-frequency noise. Each repetition was extracted by applying the peak detection algorithm, in order to synchronise the data. Correlation in time domain [32] and Magnitude Squared Coherence (MSC) in frequency domain [33] between acceleration, acceleration without gravity and angular velocities of SpaceSens sensors and Xsens MTw Awinda were then computed. The significance level of test was set at p<0.05.

#### Classification performance

Accuracy (1), sensitivity (2), specificity (3), and precision values (4), were used to evaluate the classification performance and select the best classifier; the same evaluation was carried out with real-time monitoring test.

$$\begin{array}{c} Accuracy=\frac{\sum_{k=1}^{6}\frac{TP_{k}+TN_{k}}{TP_{k}+TN_{k}+FP_{k}+FN_{k}}}{6} \end{array}$$ (1)

$$\begin{array}{c} Specificity=\frac{\sum_{k=1}^{6}\frac{TN_{k}}{TN_{k}+FP_{k}}}{6} \end{array}$$ (2)

$$\begin{array}{c} Sensitivity=\frac{\sum_{k=1}^{6}\frac{TP_{k}}{TP_{k}+FN_{k}}}{6} \end{array}$$ (3)

$$\begin{array}{c} Precision=\frac{\sum_{k=1}^{6}\frac{TP_{k}}{TP_{k}+FP_{k}}}{6} \end{array}$$ (4)

## Results

### Sensor validation

Table 1 shows the values of R and MSC of the four participants involved in the sensor validation test. SpaceSens output were compared with Xsens MTw Awinda output considering acceleration (Acc), acceleration without gravity (0g Acceleration) and angular velocity (Ang Vel) signals. Each value was obtained by averaging the R and the MSC obtained from the six sensors put on the body. Mean ± standard deviation for each signal are reported.

**Table 1:** Sensor validation results.

	Correlation			Magnitude Squared Coherence		
Subject	Acc	0g Acc	Ang Vel	Acc	0g Acc	Ang Vel
*S*01	0.97	0.905	0.968	0.949	0.744	0.963
*S*02	0.955	0.802	0.955	0.947	0.761	0.960
*S*03	0.954	0.871	0.953	0.959	0.744	0.963
*S*04	0.972	0.664	0.922	0.957	0.786	0.956
*Mean* ± *SD*	0.96 ± 0.01	0.81 ± 0.11	0.95 ± 0.02	0.95 ± 0.01	0.79 ± 0.02	0.96 ± 0.003

Linear Correlation Coefficient (R) and Magnitude Squared Coherence(MSC) between signals collected with commercial IMUs and the SpaceSens.

R values of 0.96 for acceleration, 0.81 for 0g acceleration, and 0.95 for angular velocity, representing the average respect all sensors and all subjects, are clear indicators of a very high correlation. MSC values of 0.95 for acceleration, 0.79 for simulated 0g acceleration, and 0.96 for angular velocity, far above the threshold of 0.5, make us understand that the signals are coherent and comparable, and the validation results can be considered satisfactory. Lower values of 0g Acceleration, obtained by subtracting the gravity component with the procedure explained in Signal processing section, are mostly due to the different algorithms used to estimate the orientation of the sensors: a proprietary Kalman Filter for Xsens MTw Awinda and the Mahony sensor fusion algorithm for SpaceSens. However, all the results obtained are indicators of a general condition of high correlation and coherence among the signals under analysis. In the end, low standard deviation suggests that inter-subject variability is negligible.

### Classification

The original feature dataset, obtained from the 21 subjects wearing Xsens MTw Awinda during the two sessions, were composed by 1029 repetitions in row and 2250 features in column. After RFE, it was reduced in 1014 rows and 769 columns and the observations were almost evenly spread into 6 classes (184 observations in CO, 148 in KOT, 162 in VK, 175 in RB, 161 in RH, 184 in SH).

As already mentioned, each classifier was trained considering a portion equal to 70% of observations and then tested with the remained 30% of data. Table 2 shows the results obtained.

**Table 2:** Classification results.

	Train			
Classifier	Accuracy	Specificity	Sensitivity	Precision
**DT**	89.03%	93.45%	66.79%	67.49%
**RF**	93.02%	95.82%	78.22%	78.08%
**KNN**	97.72%	98.65%	92.76%	93.01%
**SVM**	98.15%	98.91%	94.04%	94.09%
**MLP**	98.29%	98.99%	94.85%	94.67%

Performance of the classifiers in terms of accuracy, specificity, sensitivity and precision, during training and testing phase of data collected with Xsens MTw Awinda.

All algorithms tested achieved high accuracy. DT and RF showed lowest values of sensitivity and precision, so they were excluded from the selection. KNN was more sensitive and precise, but the prediction is thought to be less stable and more subjected to outliers. Finally, SVM and MLP are the classifiers with the best performance. The final choice was MLP since it showed the best results. Furthermore, it is able to extract the percentage of the observations belonging to each class by using a Softmax layer. This could be used to provide multiple corrective advices in case of mixed errors during the exercise execution, quite common in the classes of errors analysed (e.g.: bringing the knees over the toes together with raising the heels during squatting).

### Real-time system testing

The data collected from the 12 participants during the second session included a total of 319 repetitions spread in the 6 classes as follow: 30 for CO, 35 for KOT, 34 for VK, 30 for RB, 30 for RH and 29 for SH.

The classifier showed accuracy, specificity, sensitivity and precision values of 89.03%, 93.35%, 66,77%, 68,81% respectively.

Table 3 shows the confusion matrix of the real-time classification in which a trend to misclassify KOT as RH and VK as KOT is clear, that requires combined corrective feedback instructions thanks to the Softmax Layer.

**Table 3:** Confusion matrix of real-time classification.

	**CO**	**KOT**	**VK**	**RB**	**RH**	**SH**
**CO**	27	9	1	3	1	5
**KOT**	3	39	10	0	9	1
**VK**	0	8	27	0	4	6
**RB**	5	3	0	40	3	2
**RH**	2	15	1	0	41	0
**SH**	5	4	0	3	2	40

The table shows the confusion matrix of the real-time exercise execution classification: the predicted values are organised by rows and the actual values by columns. List of abbreviations: correct observation (CO), knees overcoming toes (KOT), valgus knees (VK), rounded back (RB), raised heels (RH) and shallow squat (SH).

## Discussion

The aim of the present study was to develop a wearable, real time, IMU-based biofeedback system to monitor the training process that astronauts undergo during long duration space flights to counter the effects of the microgravity on their health. The system comprises six IMUs, the SpaceSens, collecting data synchronously and sending them via Bluetooth to a software application running on a personal computer that processes signals to simulate microgravity, to extract a single repetition and to classify exercises execution. The tool can be operated through a user-friendly software interface that also displays the exercises quality classification in real-time.

Multiple algorithms were compared in order to obtain the best classification outcome, and each of them showed high accuracy. Among those, the best performance were obtained using a MLP, with an accuracy that surpassed that of other studies available in literature [24, 25]. Moreover, thanks to Softmax layer, the MLP was able to recognise multiple mixed errors during exercise execution by assigning the percentage probability with which an instance belongs to the different classes.

While the results are surely satisfactory, our study present some limitations and future perspectives to work on. The underlying hypothesis of on-ground and in-flight exercise kinematics equivalence shall be validated by collecting data from astronauts performing exercises during space flights and compared them with on-ground subject performances. Due to ethical reasons, all of the subjects participating in the study were expert athletes to avoid potential health risks related to wrong exercise execution: such constraint may have produced a bias that could potentially affect amateurs subjects (e.g.: slightly incorrect exercises may be classified as totally wrong).

A strong limitation of the study has been the organisational difficulty in carrying out the procedure needed for acquiring the subject data, constraining the dimension of the dataset.

## Conclusion

In this work, a microgravity-compatible system for real-time training monitoring of squat was developed using six IMUs and supervised machine learning algorithms. Signal collected with SpaceSens sensors showed high correlation and magnitude squared coherence with signals obtained with the gold standard MTw Xens. Thus, the system can be considered reliable to collect inertial data. Among the classifiers tested, the MLP showed the best performance, being able to classify squat techniques in real-time among six different classes (one related to correct executions and five to mistakes) with an accuracy of 89.03%. In the end, the algorithm developed to estimate orientation and subtract gravitational accelerations was effective to simulate microgravity conditions, thus, it is expected to perform well both on ground and during space missions.

## Supporting information

S1 Checklist STROBE statement—Checklist of items that should be included in reports of observational studies. (DOCX)
