# Supplementary material for: IMU-based classification of resistive exercises for real-time training monitoring on board the international space station with potential telemedicine spin-off
Source: PLoS One. 2023 Aug 10;18(8):e0289777. doi: 10.1371/journal.pone.0289777 (PMC10414632; doi:10.1371/journal.pone.0289777)
Supplement: S1 Checklist — (DOCX) [file pone.0289777.s001.docx]

STROBE Statement—checklist of items that should be included in reports of observational studies

|  | Item No. | Recommendation | Page  No. | Relevant text from manuscript |
| --- | --- | --- | --- | --- |
| **Title and abstract** | 1 | (*a*) Indicate the study’s design with a commonly used term in the title or the abstract | 1 (abstract) | “A cross-sectional observational study was conducted […] ” |
|  |  | (*b*) Provide in the abstract an informative and balanced summary of what was done and what was found | 1 (abstract) | “A cross-sectional observational study was conducted to (i) compare the signals acquired with our system to those obtained with the current state-of-the-art inertial sensors and (ii) to assess the exercises classification performance. […] signals collected with the two different systems shows good agreement between the data […] The resulting system represents a novel low-cost training monitor tool […]” |
| Introduction | | | |  |
| Background/rationale | 2 | Explain the scientific background and rationale for the investigation being reported | 2 | “[…] prolonged exposure to microgravity [..] is responsible for several physiological alterations affecting mostly the cardio-vascular and musculoskeletal systems. […] To reduce these health problems, several countermeasure training programs have been implemented [based on] feedback from on ground specialists by using a real-time audio/video system […] loss of communication can occur. […] To overcome reduced opportunity for human coaching, the introduction of motion tracking technologies could be useful.” |
| Objectives | 3 | State specific objectives, including any prespecified hypotheses | 2 | “ […] design a wearable IMU system and to develop and validate an algorithm for classifying resistance training.” |
| Methods | | | |  |
| Study design | 4 | Present key elements of study design early in the paper |  |  |
| Setting | 5 | Describe the setting, locations, and relevant dates, including periods of recruitment, exposure, follow-up, and data collection | 4 | “The data collection sessions were conducted in March and July 2021, in two different equipped gyms: one in Pavia (PV), Italy and the other Bresso (MI), Italy. Subjects were recruited on the spot on a voluntary basis, and were handed a recruitment form to sign before starting the exercises. Data was only collected in real-time during the exercises execution, and stored with a subject-specific ID (to allow anonimization while retrieving salient anagraphical information such as gender, height and weight).” |
| Participants | 6 | (*a*) *Cohort study*—Give the eligibility criteria, and the sources and methods of selection of participants. Describe methods of follow-up  *Case-control study*—Give the eligibility criteria, and the sources and methods of case ascertainment and control selection. Give the rationale for the choice of cases and controls  *Cross-sectional study*—Give the eligibility criteria, and the sources and methods of selection of participants | 4 | “recruiting […] subjects with the following inclusion criteria: healthy, trained, with previous experience of weightlifting, with no musculoskeletal injuries and aware of their strength capacity, computed with the ‘one repetition maximum’ test.” |
|  |  | (*b*) *Cohort study*—For matched studies, give matching criteria and number of exposed and unexposed  *Case-control study*—For matched studies, give matching criteria and the number of controls per case | Not applicable |  |
| Variables | 7 | Clearly define all outcomes, exposures, predictors, potential confounders, and effect modifiers. Give diagnostic criteria, if applicable | Not applicable |  |
| Data sources/ measurement | 8* | For each variable of interest, give sources of data and details of methods of assessment (measurement). Describe comparability of assessment methods if there is more than one group | 4 | “A low pass 6th order Butterworth filter was applied [on the raw data collected from each sensor] […]. Then, a peak and valley detection algorithm was applied […]. For each of the six sensors, two dataset tables were created […]. For each of the six sensors, two dataset tables were created, one containing x, y and z 0g accelerations entries and another including the corresponding components of angular velocity.” |
| Bias | 9 | Describe any efforts to address potential sources of bias | Not applicable |  |
| Study size | 10 | Explain how the study size was arrived at |  | “The total number of recruited participants is in accordance to similar studies on movement analysis with motion capture systems during physical exercises” |

Continued on next page

| Quantitative variables | 11 | Explain how quantitative variables were handled in the analyses. If applicable, describe which groupings were chosen and why | 5 | “Features in time and frequency domains were computed from the segmented signals of each sensor […]. Feature scaling was then performed by using a Robust Scaler and the dataset was reduced to increase efficiency of the classifier by applying the Recursive Feature Elimination (RFE) method […]. The ideal number was chosen by applying RFE multiple times and considering the one that permitted to reach the best results of the classification. The resulting dataset was then used to train and test the classifiers, considering 70% and 30% of all the values respectively.” |
| --- | --- | --- | --- | --- |
| Statistical methods | 12 | (*a*) Describe all statistical methods, including those used to control for confounding | 5 | “We compared signals [from SpaceSens and Xsens sensors]. […] Correlation in time domain and Magnitude Squared Coherence (MSC) in frequency domain between acceleration, acceleration without gravity and angular velocities of SpaceSens sensors and Xsens […] were computed. The significance level of test was set at p < 0.05.” |
|  |  | (*b*) Describe any methods used to examine subgroups and interactions |  | Not applicable |
|  |  | (*c*) Explain how missing data were addressed | 4 | ”Incomplete data from a set of exercise repetitions (i.e., loss of data due to sporadic sensor communication malfunctioning) were discarded prior to further statistical analysis.” |
|  |  | (*d*) *Cohort study*—If applicable, explain how loss to follow-up was addressed  *Case-control study*—If applicable, explain how matching of cases and controls was addressed  *Cross-sectional study*—If applicable, describe analytical methods taking account of sampling strategy |  | Not applicable |
|  |  | (*e*) Describe any sensitivity analyses | 5 | “Five supervised machine learning methods were compared […]. Each algorithm was trained and tested with the same subset of data and their performances were evaluated by a stratified 10-fold Cross-Validation. Accuracy, sensitivity, specificity, and precision values, were used to evaluate the classification performance and select the best classifier; the same evaluation was carried out with real-time monitoring test.” |
| Results | | | | |
| Participants | 13* | (a) Report numbers of individuals at each stage of study—eg numbers potentially eligible, examined for eligibility, confirmed eligible, included in the study, completing follow-up, and analysed |  | Not applicable |
|  |  | (b) Give reasons for non-participation at each stage |  | Not applicable |
|  |  | (c) Consider use of a flow diagram |  | Not applicable |
| Descriptive data | 14* | (a) Give characteristics of study participants (eg demographic, clinical, social) and information on exposures and potential confounders |  | Not applicable |
|  |  | (b) Indicate number of participants with missing data for each variable of interest |  | Not applicable |
|  |  | (c) *Cohort study*—Summarise follow-up time (eg, average and total amount) |  | Not applicable |
| Outcome data | 15* | *Cohort study*—Report numbers of outcome events or summary measures over time |  | Not applicable |
|  |  | *Case-control study—*Report numbers in each exposure category, or summary measures of exposure |  | Not applicable |
|  |  | *Cross-sectional study—*Report numbers of outcome events or summary measures |  | Not applicable |
| Main results | 16 | (*a*) Give unadjusted estimates and, if applicable, confounder-adjusted estimates and their precision (eg, 95% confidence interval). Make clear which confounders were adjusted for and why they were included |  | Not applicable |
|  |  | (*b*) Report category boundaries when continuous variables were categorized | 6 | “[The MLP classifier is] able to extract the percentage of the observations belonging to each [categorical] class by using a Softmax layer […] to provide multiple corrective advices in case of mixed errors during the exercise execution” |
|  |  | (*c*) If relevant, consider translating estimates of relative risk into absolute risk for a meaningful time period |  | Not applicable |

Continued on next page

| Other analyses | 17 | Report other analyses done—eg analyses of subgroups and interactions, and sensitivity analyses | 6 | “R values […] representing the average respect all sensors and all subjects, are clear indicators  of a very high correlation. MSC values [show that] the signals are coherent and comparable, and the validation results can be considered satisfactory […] All algorithms tested achieved high accuracy. DT and RF showed lowest values of sensitivity and precision […]. KNN was more sensitive and precise, but the prediction is thought to be less stable and more subjected to outliers. Finally, SVM and MLP are the classifiers with the best performance. The final choice was MLP since it showed the best results” |
| --- | --- | --- | --- | --- |
| Discussion | | | | |
| Key results | 18 | Summarise key results with reference to study objectives |  | “The aim of the present study was to develop a wearable, real time, IMU-based biofeedback system to monitor training […].  The system comprises six IMUs, collecting data synchronously and sending them via Bluetooth to a personal computer in which signals are processed […] to extract a single repetition and to perform the classification among correct and wrong execution. The tool can be operated through a user-friendly software interface that also displays the quality of the exercises execution classification in real-time.” |
| Limitations | 19 | Discuss limitations of the study, taking into account sources of potential bias or imprecision. Discuss both direction and magnitude of any potential bias | 7/8 | “The underlying hypothesis of on-ground and in-flight exercise kinematics equivalence shall be validated by collecting data from astronauts performing exercises during space flights and compared them with on-ground subject performances.  Due to ethical reasons, all of the subjects participating in the study were expert athletes to avoid potential health risks related to wrong exercise execution: such constraint may have produced a bias that could potentially affect amateurs subjects (e.g.: slightly incorrect exercises may be classified as totally wrong). A strong limitation of the study has been the organizational difficulty in carrying out the procedure needed for acquiring the subject data, constraining the dimension of the dataset.” |
| Interpretation | 20 | Give a cautious overall interpretation of results considering objectives, limitations, multiplicity of analyses, results from similar studies, and other relevant evidence | 8 | “[…] the system can be considered reliable to collect inertial data. Among the classifiers tested, the Multi-Layer Perceptron showed the best performances. It was able to real-time classify squat techniques in six different classes, one related to correct executions and five to mistakes, with an accuracy of 89.03%. In the end, the algorithm developed to estimate orientation and subtract gravitational accelerations was effective to simulate microgravity conditions, thus, it is expected to perform well both on ground and during space missions.” |
| Generalisability | 21 | Discuss the generalisability (external validity) of the study results | 8 | “Indeed, while the intended scope of the tool is to monitor astronauts training (albeit its development was carried out completely on ground), such portable IMU system could also have a potential extended application for individual training or rehabilitation at home, allowing to perform resistive exercises safely and without local human supervision.” |
| Other information | |  | | |
| Funding | 22 | Give the source of funding and the role of the funders for the present study and, if applicable, for the original study on which the present article is based | 8 | “This work has been partially funded by ASI (Italian Space Agency), grant n°DC-VUM-2017-006 MARS-PRE” |

*Give information separately for cases and controls in case-control studies and, if applicable, for exposed and unexposed groups in cohort and cross-sectional studies.

**Note:** An Explanation and Elaboration article discusses each checklist item and gives methodological background and published examples of transparent reporting. The STROBE checklist is best used in conjunction with this article (freely available on the Web sites of PLoS Medicine at http://www.plosmedicine.org/, Annals of Internal Medicine at http://www.annals.org/, and Epidemiology at http://www.epidem.com/). Information on the STROBE Initiative is available at www.strobe-statement.org.
